# Environmental conditions, diel period, and fish size influence the horizontal and vertical movements of red snapper

**DOI:** 10.1038/s41598-021-88806-3

**Published:** 2021-05-05

**Authors:** Nathan M. Bacheler, Kyle W. Shertzer, Brendan J. Runde, Paul J. Rudershausen, Jeffrey A. Buckel

**Affiliations:** 1 Southeast Fisheries Science Center, National Marine Fisheries Service, Beaufort, NC 28516 USA; 2 Department of Applied Ecology, Center for Marine Sciences and Technology, North Carolina State University, Morehead City, NC 28557 USA

**Keywords:** Animal migration, Ocean sciences, Marine biology

## Abstract

Most demersal fishes are difficult to observe and track due to methodological and analytical constraints. We used an acoustic positioning system to elucidate the horizontal and vertical movements of 44 red snapper (*Lutjanus campechanus*) off North Carolina, USA, in 2019. Mean movement rate and distance off bottom varied by individual, with larger red snapper generally moving faster and spending more time farther off the bottom than smaller individuals. We used generalized additive mixed models that accounted for temporal autocorrelation in the data to show that mean hourly red snapper movement rate was lower during the day than at night and was negatively related to bottom water temperature. Moreover, red snapper spent more time off the bottom during the day than at night, and vertical movements were mostly related to bottom upwelling events that sporadically occurred in May–July. Our results and previous observations suggest that red snapper feed primarily on benthic organisms at night, and display diel vertical migration (i.e., thermotaxis) up to warmer waters (when present) during the day to aid digestive efficiency. Movement is a central organizing feature in ecology, and the sustainable management of fish will benefit from a better understanding of the timing and causes of fish movement.

## Introduction

The movement of organisms is a key feature of life. Most organisms move in some or all life stages, either passively or actively, and these movements influence gene flow, colonization and extinction rates, the spread of diseases, and the dynamics of populations, communities, and ecosystems^1^. Identifying when, where, why, and how marine fishes move can greatly improve their management by, for instance, elucidating the spatial and temporal patterns of habitat use^2^, optimizing marine reserve design^3^, incorporating spatial structure in fish stock assessments^4^, and improving indices of abundance^5^. Yet methods for quantifying and describing the movement of organisms have lagged^6^, perhaps owing to the multidimensional complexities of movement and substantial variability among individuals^7^. This is especially true of marine fishes that spend their lives far from shore or in deep seafloor habitats, where they are generally difficult to observe^8^.

Horizontal movements of fish are influenced by many variables including food availability, predator avoidance, water temperature, conspecific density, fish size or age, and water currents^9–14^. Vertical movement of fish has received less attention than horizontal movement. Fish have been shown to move vertically over daily time scales (i.e., diel vertical migration) for a variety of reasons including feeding, predator or competitor avoidance, reproduction, or various physiological advantages^15–19^. For example, Sims et al.^16^ showed that adult male dogfish (*Scyliorhinus canicula*) moved into warmer, shallow prey-rich areas to feed at night, and then retreated back into deeper, cooler waters to rest and digest during the day. In addition to diel movements, some species of highly migratory fishes displayed changes in depth in ways that appeared to be unrelated to diel cycles^20,21^. For instance, Kitagawa et al.^21^ showed that young Pacific bluefin tuna (*Thunnus orientalis*) undertook periodic dives to feed below the thermocline during seasons when prey was limited near the surface, but they took multiple, short dives instead of fewer, longer dives to maintain a high internal body temperature. Taylor et al.^22^ documented juvenile anchovies (*Anchoa* spp.) making brief downward forays into hypoxic hypolimnetic waters that contained high copepod concentrations. Some species of reef fishes display vertical movements, but most studies have only used fish depth as an indicator of their habitat use (e.g., Ref.^23^).

**Figure 1:**
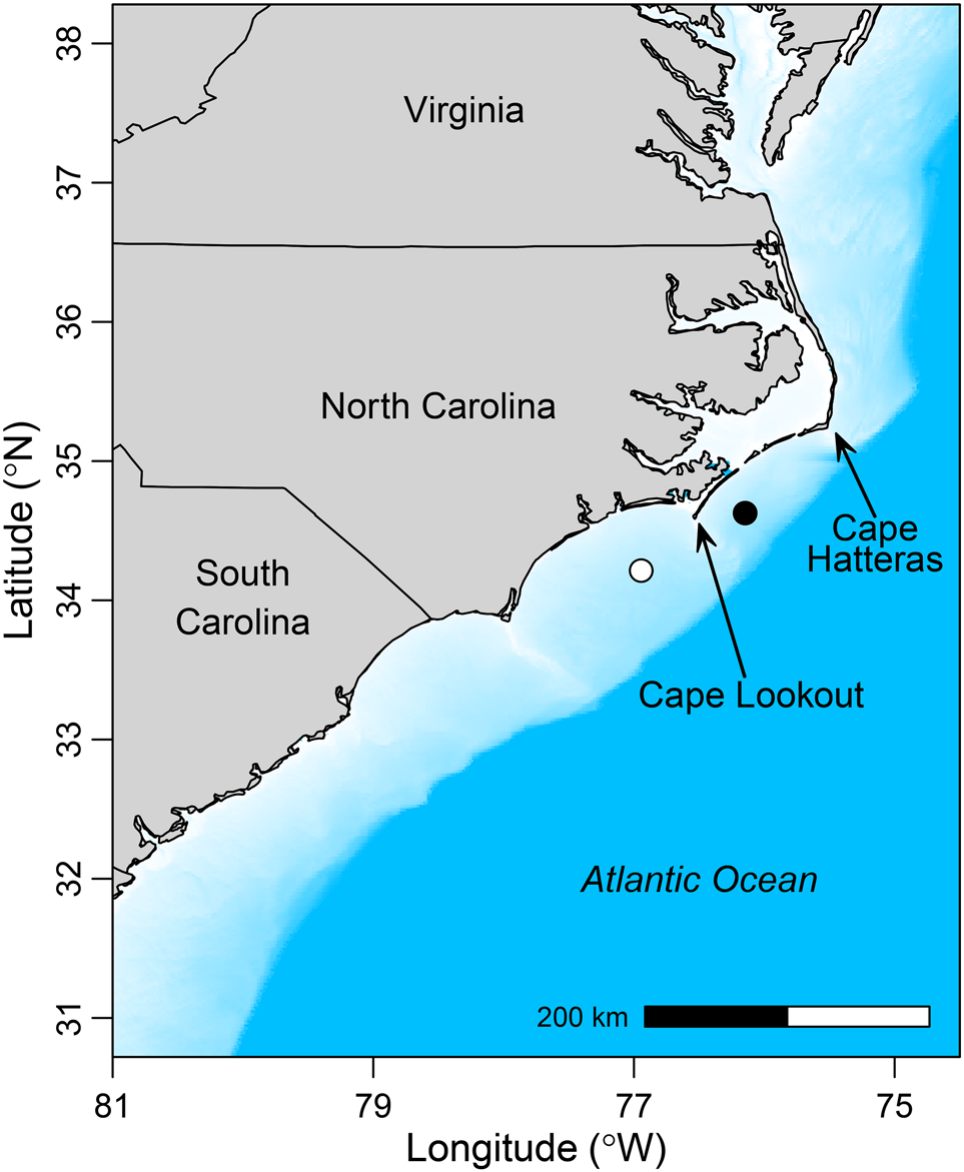
Location of the red snapper (*Lutjanus campechanus*) tracking study (filled black circle) between Cape Hatteras and Cape Lookout, North Carolina, USA, in 2019. Some environmental data used in this study came from NOAA buoy 41159, the location of which is indicated by the filled white circle. Water depth is shown in blue (lightest blue < 5 m; darkest blue > 100 m).

Red snapper (*Lutjanus campechanu*s) is a reef-associated fish species that displays complex horizontal and vertical movement behaviors. This species is found along the southeast United States Atlantic coast (hereafter, SEUS; Fig. 1), the Gulf of Mexico, and the northeastern coast of South America. Historically, red snapper have been heavily targeted throughout their distribution, and now they are strictly managed as a recreational and commercial species^24^. The SEUS fishery for red snapper has been closed or only has had a small catch allowance since 2010 due to its overfished status^25^. Tagging and telemetry studies in the Gulf of Mexico have shown that red snapper closely associate with artificial and natural structured habitats and display fairly high site fidelity^26–28^. Red snapper are typically considered a benthic reef fish species, but Williams-Grove and Szedlmayer^29^ showed that they spend considerable time off the seafloor in spring and summer months, sometimes even reaching the surface; the authors noted that the reasons for these vertical movements are unknown but could include summertime spawning activity^30^, avoidance of hypoxic bottom waters, pelagic prey availability, or attraction to vessels. Williams-Grove and Szedlmayer^29^ used a reference transmitter to estimate the accuracy and precision of fish positions and we do the same here.

We quantify and attempt to explain the horizontal and vertical movement behaviors of adult^30^ red snapper using a fine-scale acoustic telemetry positioning system in the SEUS. There were three primary objectives of our work. The first was to determine if our acoustic telemetry positioning system provided positional data for red snapper that were precise and accurate enough to reliably quantify fine-scale movements. The second objective was to test for individual variability in movement rate and distance off bottom for acoustically tagged red snapper, and if individual variability was present, determine if fish size could help explain that variability. The last objective was to make inferences about the correlates of horizontal movements of red snapper and why they periodically swim up into the water column. We assessed this objective by relating hourly red snapper movement rates or distances off the bottom to a suite of environmental variables using generalized additive mixed models. The environmental variables chosen were based on prior studies on other species (e.g., time of day) or hypotheses previously developed for red snapper (e.g., avoidance of bottom water).

## Results

**Figure 2:**
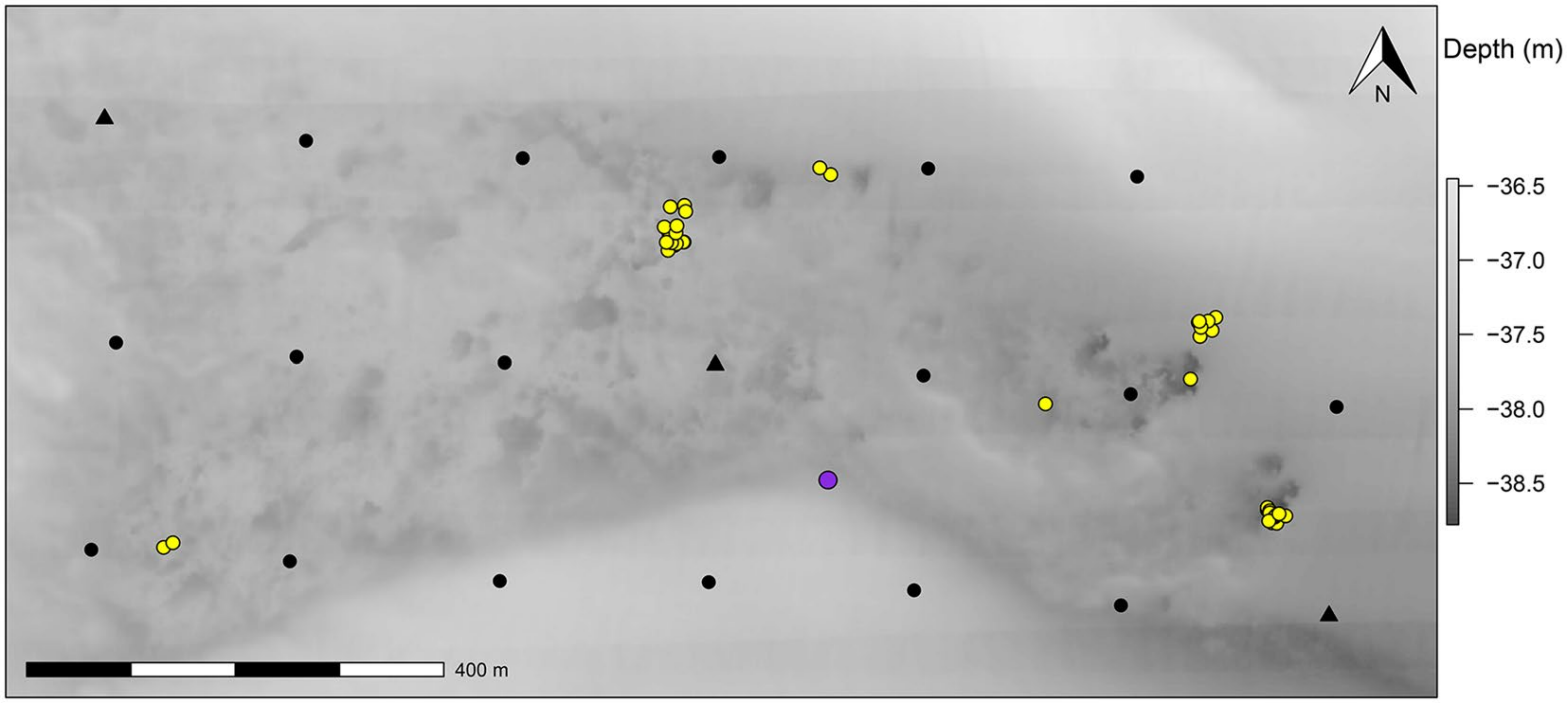
Study area (~ 34°N, 76°W) of the red snapper (*Lutjanus campechanus*) telemetry positioning system at the Chicken Rock area of Raleigh Bay, North Carolina, in 2019. Water depth is indicated by the background map, with lighter gray being shallower than darker gray. Underwater receivers are indicated by filled black circles, except for receivers with attached current probes, which are shown by filled black triangles. The reference tag location is shown by the filled purple circle, and tagging locations are indicated by filled yellow circles.

Our study at the “Chicken Rock” in Raleigh Bay, North Carolina, USA (Fig. 2; ~ 37-m deep), began on 7 May 2019 and ended on 16 December 2019. A total of 44 adult red snapper were externally tagged in our study, ranging from 390 to 860 mm total length (mean = 667 mm; sd = 113 mm; Table 1). Two fish (both tagged on 7 May 2019) retained their transmitters, survived, and remained in the study area until the study’s completion. The remaining fish experienced tag loss (*N* = 22), permanently emigrated from the study area (*N* = 15), appeared to have been preyed upon shortly after tagging (*N* = 4), or were removed by fishers (*N* = 1). There were 9 acoustically tagged red snapper that did not meet the minimum sample size requirements of 100 positional detections, so those fish were excluded from all analyses, leaving 35 fish that were included in our analyses (Table 1); those 35 fish were considered “at large” (i.e., available for detection) until tag loss, permanent emigration, predation, or harvest. There were 346,291 positional estimates from these 35 fish (mean = 9,894; sd = 10,438) that ranged in size from 410 to 860 mm total length (mean = 711 mm; sd = 97). These acoustically tagged red snapper were detected from 4 to 220 unique days of the study (mean = 67; sd = 48), while days at large ranged from 4 to 224 days (mean = 86; sd = 51). The residency index, which was calculated as the number of days detected divided by the number of days at large for each fish, ranged from 0.28 to 1.00 (mean = 0.79; sd = 0.25; Table 1).

**Table 1:** Information for individual red snapper (*Lutjanus campechanus*) tagged at the Chicken Rock area in Raleigh Bay, North Carolina, USA, in 2019.

Fish	Tag	Length (mm TL)	Date tagged	Last day detected	Number of positions observed	Number of days detected	Days at large	Residency index	Fate	Included in analyses?
1	4290	520	7-May	–	0	0	0	–	Tag loss	No
2	4291	700	7-May	15-Sep	18,814	121	132	0.92	Emigration	Yes
3	4292	720	7-May	28-Aug	20,785	108	114	0.95	Harvest	Yes
4	4293	685	7-May	10-May	604	4	4	1.00	Tag loss	Yes
5	4294	665	7-May	13-Aug	15,201	99	99	1.00	Tag loss	Yes
6	4295	785	7-May	5-Jun	799	17	30	0.57	Tag loss	Yes
7	4296	635	7-May	26-Jun	2755	27	50	0.54	Emigration	Yes
8	4297	680	7-May	27-Aug	16,635	113	113	1.00	Tag loss	Yes
9	4298	720	7-May	16-Dec	21,297	173	224	0.77	Alive	Yes
10	4299	750	7-May	26-May	4698	20	20	1.00	Tag loss	Yes
11	4300	740	7-May	19-Jun	1290	19	44	0.43	Tag loss	Yes
12	4301	860	7-May	1-Aug	22,384	87	87	1.00	Tag loss	Yes
13	4302	500	7-May	7-May	6	1	1	1.00	Emigration	No
14	4303	705	7-May	27-Jul	265	23	82	0.28	Emigration	Yes
15	4304	710	7-May	20-May	3005	14	14	1.00	Tag loss	Yes
16	4305	760	7-May	16-Dec	52,189	220	224	0.98	Alive	Yes
17	4306	765	7-May	29-Jul	20,316	84	84	1.00	Tag loss	Yes
18	4307	740	7-May	1-Jul	5208	50	56	0.89	Tag loss	Yes
19	4308	720	7-May	14-Jul	13,505	69	69	1.00	Tag loss	Yes
20	4309	795	7-May	29-Aug	12,427	108	115	0.94	Tag loss	Yes
21	5228	390	7-May	8-May	12	2	2	1.00	Emigration	No
22	5229	690	7-May	20-Jun	6755	41	45	0.91	Tag loss	Yes
23	5230	730	7-May	14-May	1316	8	8	1.00	Tag loss	Yes
24	5231	530	13-Aug	–	0	0	0	–	Emigration	No
25	7269	735	13-Aug	11-Nov	7979	63	91	0.69	Emigration	Yes
26	7270	750	13-Aug	25-Sep	1309	13	43	0.30	Emigration	Yes
27	7271	760	13-Aug	15-Dec	15,277	112	125	0.90	Emigration	Yes
28	7272	715	13-Aug	13-Aug	27	1	1	1.00	Tag loss	No
29	7273	735	13-Aug	16-Sep	4370	35	35	1.00	Tag loss	Yes
30	7274	750	13-Aug	21-Aug	23	4	9	0.44	Predation	No
31	7275	685	13-Aug	9-Nov	12,774	89	89	1.00	Tag loss	Yes
32	7276	425	13-Aug	12-Oct	7208	61	61	1.00	Tag loss	Yes
33	7277	790	13-Aug	30-Sep	8586	49	49	1.00	Tag loss	Yes
34	7278	520	13-Aug	13-Aug	1	1	1	1.00	Emigration	No
35	7279	695	13-Aug	13-Aug	3	1	1	1.00	Predation	No
36	7280	685	13-Aug	–	0	0	0	–	Predation	No
37	7281	750	13-Aug	2-Dec	22,483	112	112	1.00	Tag loss	Yes
38	7282	720	13-Aug	15-Dec	1766	68	125	0.54	Emigration	Yes
39	7283	775	13-Aug	15-Dec	3299	43	125	0.34	Emigration	Yes
40	7284	845	13-Aug	10-Dec	969	68	120	0.57	Emigration	Yes
41	7285	745	13-Aug	15-Dec	2324	67	125	0.54	Emigration	Yes
42	7286	755	13-Aug	27-Nov	450	33	107	0.31	Emigration	Yes
46	7994	410	30-Aug	12-Nov	10,996	73	92	0.79	Predation	Yes
49	4292	475	22-Sep	6-Nov	6253	46	86	0.53	Tag loss	Yes

Fish is the fish number, Tag is the transmitter number, Last day detected was the last day the fish was detected at the study area alive, Residency index is the number of unique days detected divided by the number of days at large, and Fate is the suspected fate of each fish determined from position data (Alive = alive and in study area at end of study). Only fish with 100 or more observed positions were included in the analyses.

**Figure 3:**
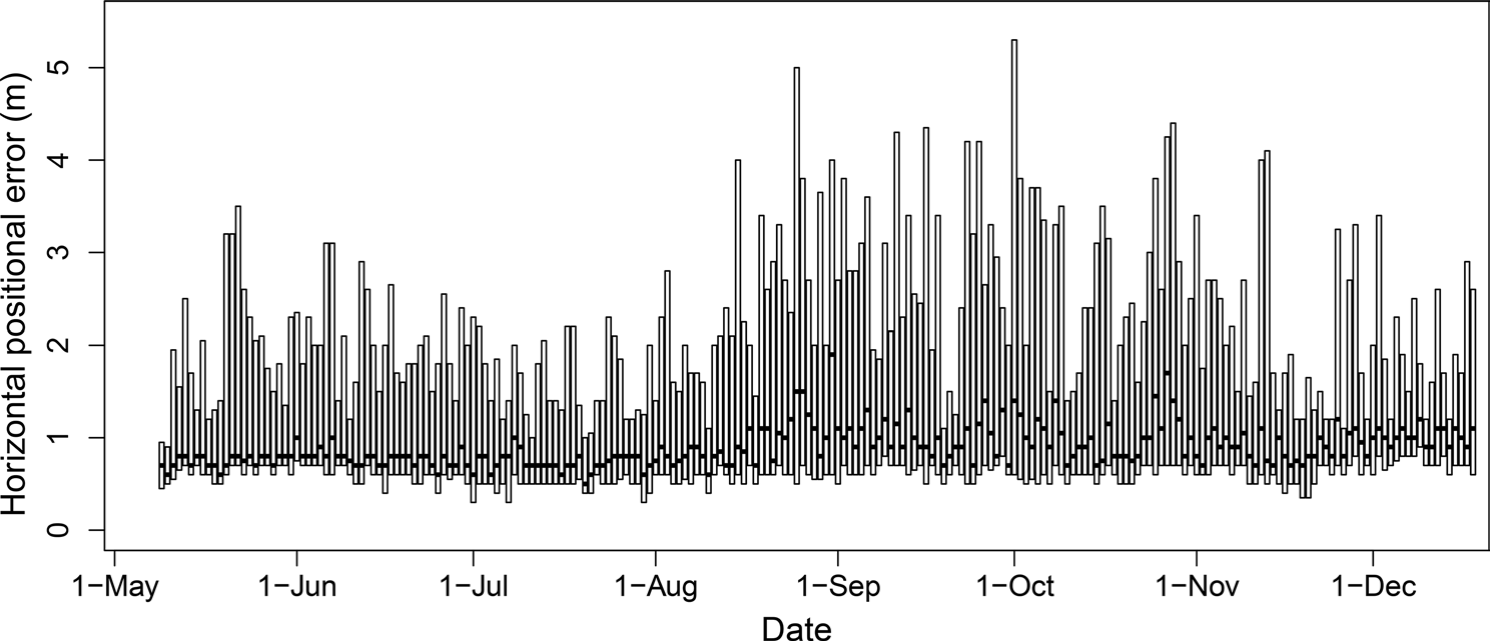
Horizontal positional error (m) of a reference transmitter deployed at the Chicken Rock area in Raleigh Bay, North Carolina, USA, in 2019. Horizontal positional error was calculated as the difference between the known position of the reference tag and its estimated position, shown with daily boxes. Median horizontal positional error is indicated by the thick horizontal black lines, and 25th and 75th percentiles are shown by the bottom and top of gray boxes.

Median horizontal positional error from 18,026 detections of the reference transmitter was 0.8 m, and daily medians ranged from approximately 0.6–2.0 m (Fig. 3). Moreover, horizontal positional error estimates were ~ 0.8 m across each binned hour of the diel period. There were some rare large horizontal positional error estimates (~ 12 m), but the vast majority of positions (> 99%) were precise and accurate, suggesting the spatial positions of acoustically tagged red snapper were likewise precise and accurate.

**Figure 4:**
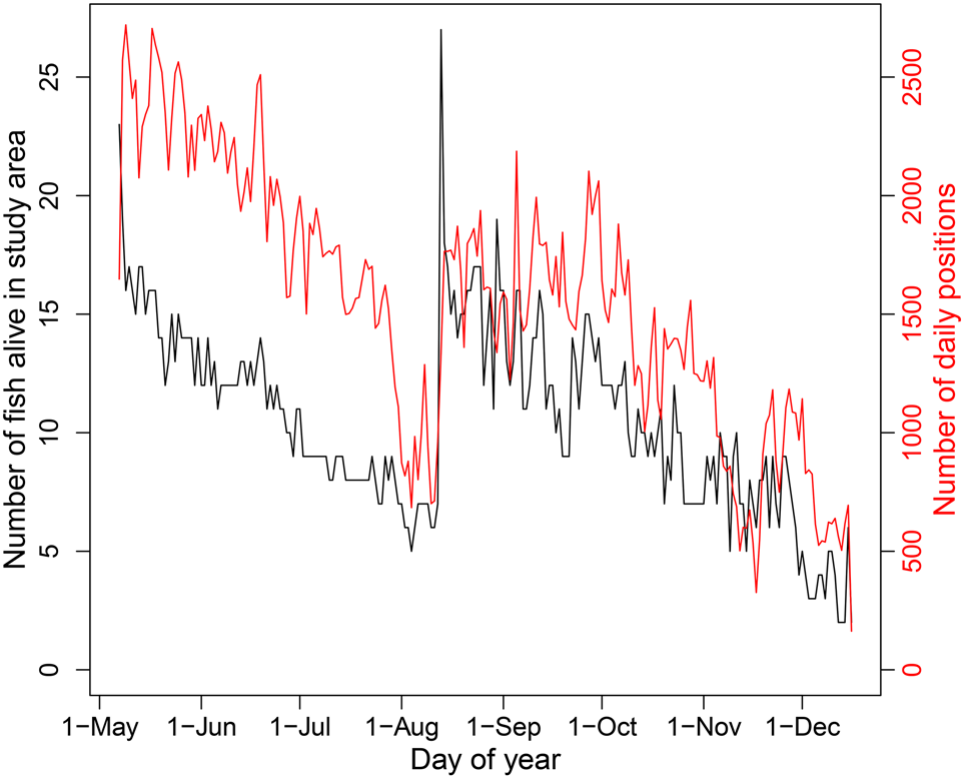
Daily number of acoustically tagged red snapper (*Lutjanus campechanus*) alive and in the study area (black), and the number of daily positions available from those fish (red), at the Chicken Rock area of Raleigh Bay, North Carolina, USA, in 2019. Twenty-three red snapper were tagged on 7 May 2019, nineteen were tagged on 13 August 2019, one was tagged on 30 August 2019, and one was tagged on 22 September 2019.

The number of acoustically tagged red snapper alive, in the study area, and included in our analyses ranged from 2 to 27 fish detected per day (daily median = 10 fish; Fig. 4). The number of acoustically tagged red snapper declined approximately linearly after each tagging event, falling from 23 at tagging on 7 May 2019 to approximately 5 fish by early August 2019. After 19 additional fish were tagged on 13 August 2019, the number of acoustically tagged fish alive in the array increased briefly to a maximum of 27. These fish disappeared at a nearly constant rate until the end of the study, when 2–6 acoustically tagged red snapper remained in the study area. The number of daily positions available from these fish mirrored the number of fish alive in the study area, and ranged from 163 (on 16 December 2019, the last day of the study) to 2720 (on 9 May 2019, the third day of the study; Fig. 4).

**Figure 5:**
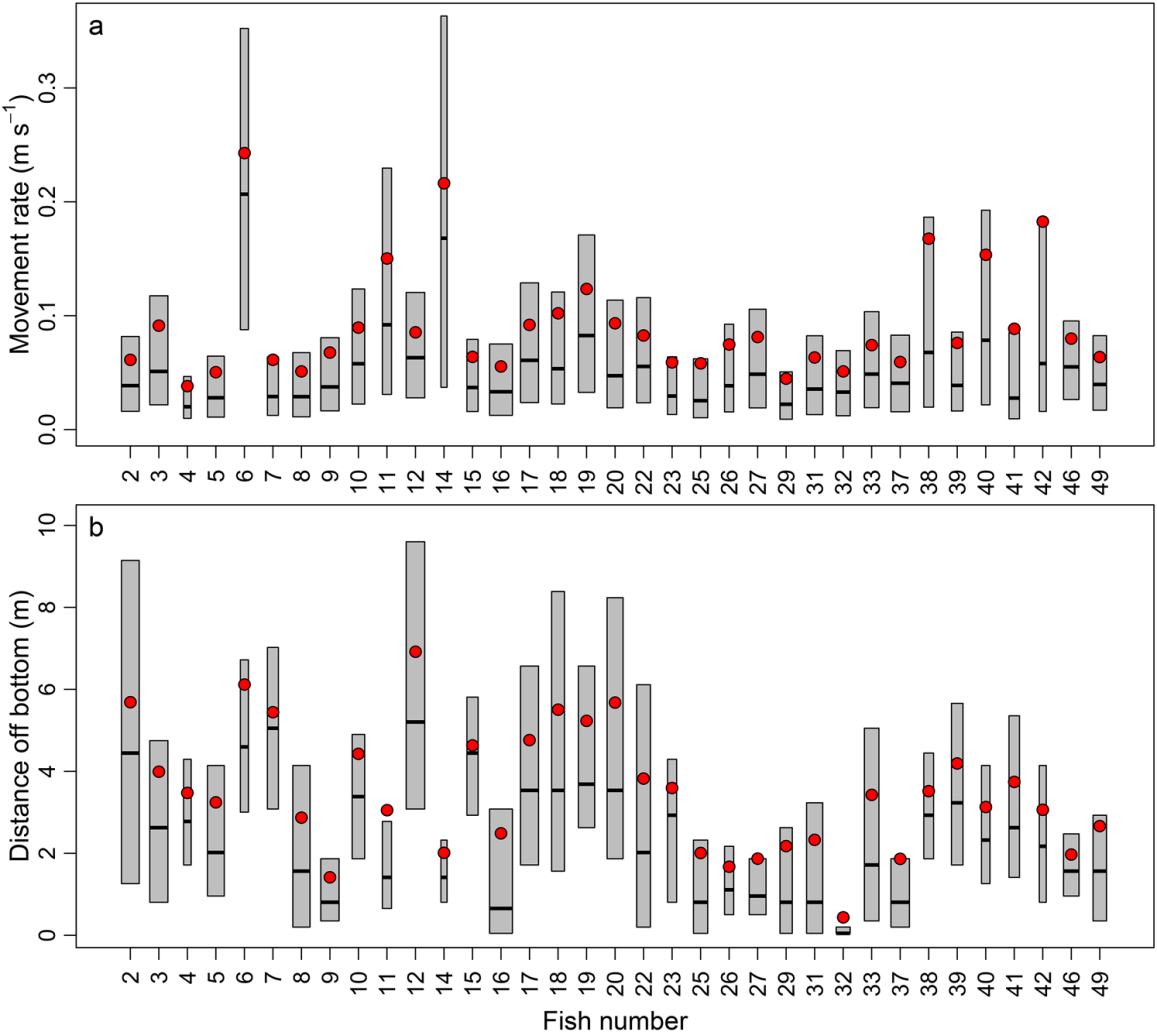
Individual-level variability in movement rates (**a**) and distance off bottom (**b**) for acoustically tagged red snapper (*Lutjanus campechanus*) at the Chicken Rock area of Raleigh Bay, North Carolina, in 2019. The thick horizontal black lines indicate medians and the top and bottom of the gray boxes show 25th and 75th percentiles; mean values for each fish are given by the filled red circles. Box width is scaled to the sample size for each fish, which ranged from 265 to 52,189 observations. Only individuals included in the analyses are shown here.

There were substantial differences in movement rates and distances off the bottom across individual acoustically tagged red snapper (Fig. 5). The median movement rate across all red snapper was 0.04 m s^−1^, but median movement rates were highly variable among individuals (linear model: *F* = 626.7, df = 34, *p* < 0.0001), ranging from 0.02 to 0.21 m s^−1^. The model including individual differences (ΔAIC = 0) performed better than models excluding the effect of individual (ΔAIC = 20,611). Generally, acoustically tagged red snapper with fewer detections appeared more likely to display higher movement rates than fish with more detections (Fig. 5). There was also significant variation in the distance off the bottom for red snapper, with models that excluded individual differences having a ΔAIC value = 59,363. The overall median distance off the bottom was 2.0 m, but ranged from 0.3 to 5.2 m among individuals (linear model: *F* = 1906.7, df = 34, *p* < 0.0001). Individual variability in red snapper movements was significant but only explained a small amount of the observed variation in movement rate (*R*^2^ = 5%) or distance off bottom (*R*^2^ = 16%).

**Figure 6:**
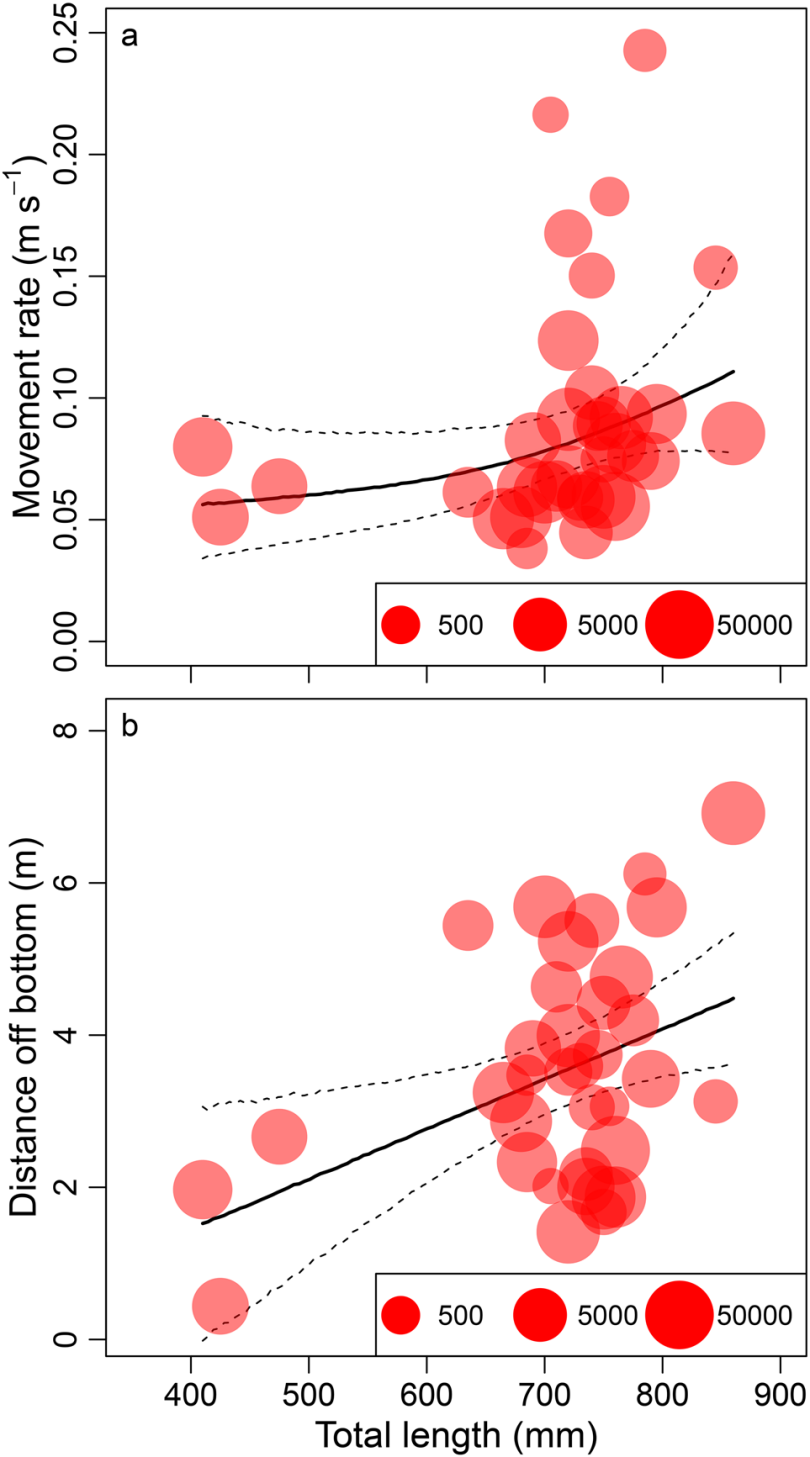
Relationships between red snapper (*Lutjanus campechanus*) mean movement rates (**a**) or mean distances off the bottom (**b**) and fish total length at the Chicken Rock in Raleigh Bay, North Carolina, in 2019. Point size is scaled to the number of movement rate or distance off bottom observations for each fish, solid line is the mean fit from generalized additive models, and dashed lines are 95% confidence intervals.

Larger red snapper were more likely to move at a higher rate and be detected at greater distances off the bottom than smaller red snapper (Fig. 6). Red snapper length was included as a predictor variable when using log-transformed movement rates in generalized additive models (GAMs), as indicated by a worse ΔAIC value for models excluding length (ΔAIC = 2.1). The model including red snapper length explained 13% of the model deviance. Similar results were found for the GAM including fish length as a predictor of red snapper distance off the bottom, where the model including fish length was better than the model excluding it (ΔAIC = 4.8). Fish length explained 18% of the deviance of the distance off bottom GAM (Fig. 6).

**Figure 7:**
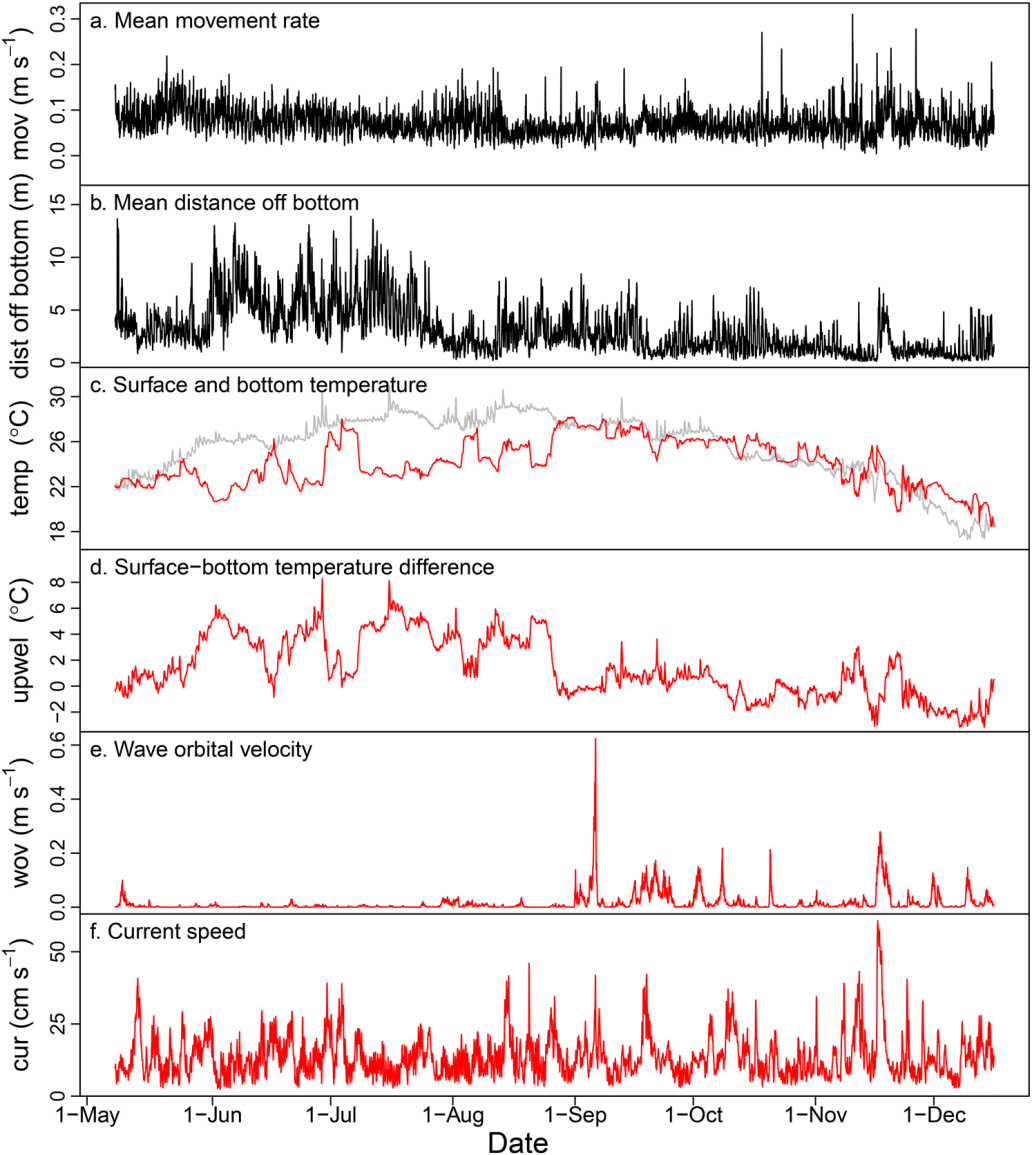
Hourly response (black lines in **a,b**) and predictor variables (red and gray lines in **c**–**f**) included in the generalized additive mixed models to explain red snapper (*Lutjanus campechanus*) movement rate and distance off bottom at the Chicken Rock area in Raleigh Bay, North Carolina, USA, in 2019. In panel **c**, surface temperature is shown in gray and bottom temperature is shown in red. Note that surface temperature and wave orbital velocity were measured from a NOAA buoy 85 km away from the study site.

Hourly response and predictor variables included in the generalized additive mixed models (GAMMs) displayed distinct patterns over the course of the study (Fig. 7). Hourly movement rates varied substantially over short time scales, while also peaking broadly in late May, early August, and November. Distance off the bottom was highest in late May through July, and lower in mid-May and August–December. Bottom temperature was highly variable, displaying rapid increases and decreases in late May through July that were also reflected in the surface to bottom temperature difference over the same time period. Wave orbital velocity was highest during Hurricane Dorian on 6 September 2019 (~ 0.6 m s^−1^), but also reached ~ 0.2 m s^−1^ during other weather events later in the fall. Current speed was also highly variable over the course of the study, appearing to vary on approximately weekly time scales (Fig. 7).

**Figure 8:**
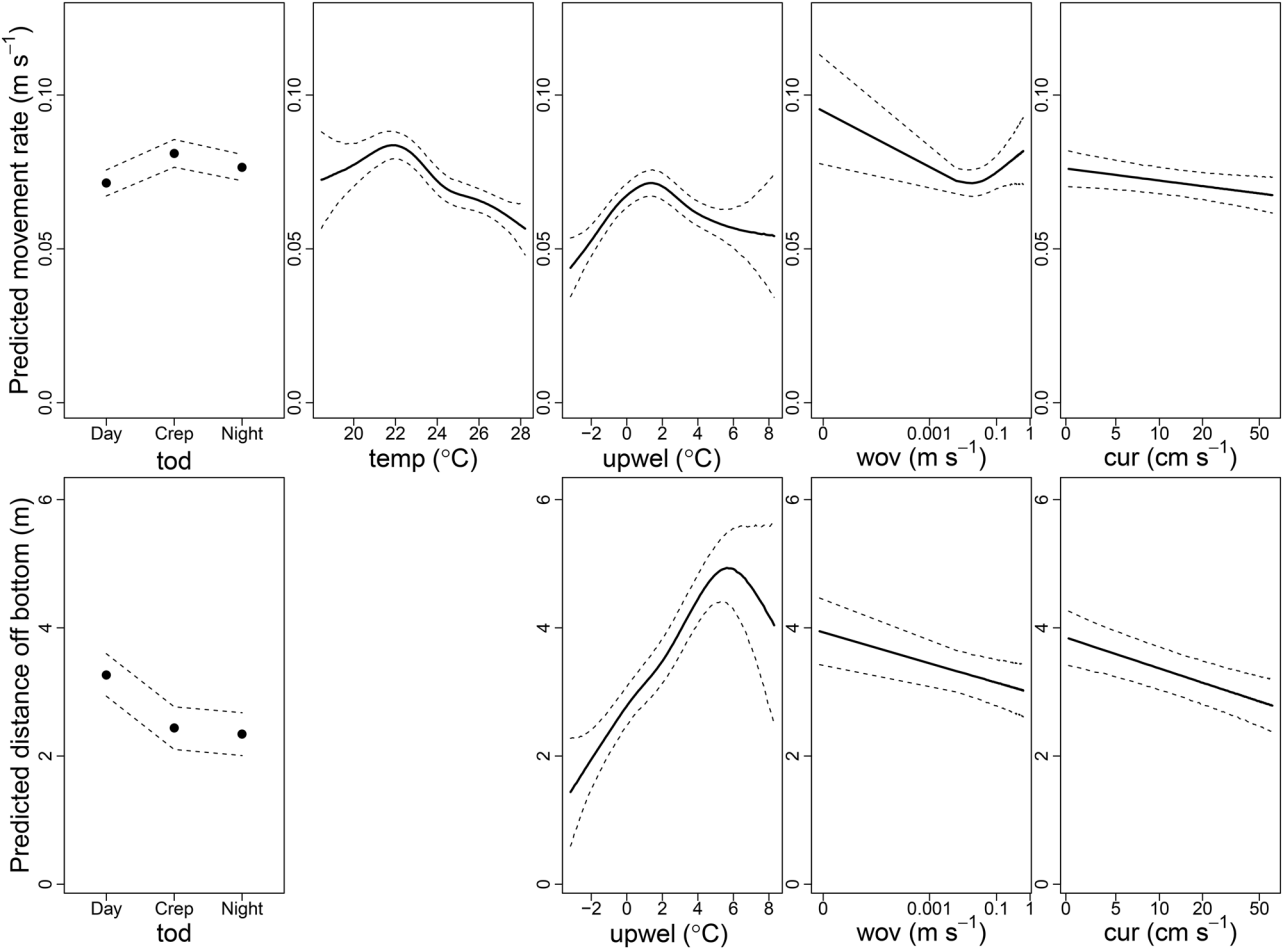
Covariate plots for the best generalized additive mixed models explaining mean red snapper (*Lutjanus campechanus*) movement rates (top row) or distance off bottom (bottom row) at the Chicken Rock area in Raleigh Bay, North Carolina, in 2019. Mean values are shown by either the filled black points or solid black line, and dashed lines indicate 95% confidence intervals. Predictor variables removed from models based on AIC values are not shown. Predictor variable names are time of day (tod), bottom temperature (temp), difference between surface and bottom temperatures (upwel), wave orbital velocity (wov), and current speed (cur).

The hourly GAMM of log-transformed red snapper movement rates included all predictor variables and explained 12% of the model deviance (Table 2). Specifically, mean movement rates were approximately 10% lower for acoustically tagged red snapper during the day than crepuscular periods or night, and were also negatively related to bottom water temperature, declining 32% as temperature increased from 22 to 28 °C (Fig. 8). Moreover, movement rates displayed a dome-shaped relationship to the upwelling index, being highest when the surface to bottom temperature difference was 0–2 °C. Last, there were weak negative relationships observed between movement rates and both wave orbital velocity and current speed.

**Table 2:** Model selection table for generalized additive mixed models relating movement rates or distance off bottom of acoustically tagged red snapper (*Lutjanus campechanus*) to five potential predictor variables: time of day (*tod*, with three levels: day, crepuscular, or night), bottom temperature (*temp*), surface to bottom temperature difference (*upwel*), wave orbital velocity (*wov*), and current speed (*cur*). Degrees of freedom are shown for factor (*f*) term, and estimated degrees of freedom are shown for smoothed terms (*s*).

Model	ΔAIC	*R*^2^	*f*(*tod*)	*s*(*temp*)	*s*(*upwel*)	*s*(*wov*)	*s*(*cur*)
**Movement rate**							
Full	0.0	12.4	2***	5.0***	4.6***	4.3**	1.0*
Full–*cur*	1.1	12.0	2***	4.8***	4.7***	2.8**	ex
Full–*wov*	3.9	11.3	2***	4.9***	4.7***	ex	1.0*
Full–*cur*–*wov*	4.6	11.1	2***	4.7***	4.8***	ex	ex
**Distance off bottom**							
Full–*temp*	0.0	29.7	2***	ex	4.4***	1.0**	1.0***
Full	2.1	32.5	2***	3.1	4.5***	1.0**	1.0***
Full–*temp*–*wov*	3.6	28.4	2***	ex	4.5***	ex	1.0***
Full–*wov*	6.5	30.9	2***	2.9	4.5***	ex	1.0***

The full model includes all predictor variables, while “Full” followed by the minus sign and a variable name indicates a model that does not include that predictor variable. The four best GAMMs based on ΔAIC are shown for each model, arranged from best to worst. Asterisks refer to *p*-values at the following levels: * = 0.05; ** = 0.01; *** = 0.001.

Four predictor variables were included in the hourly GAMM of distance off the bottom of acoustically tagged red snapper, which explained 30% of the model deviance (Table 2). The main similarity with the movement GAMM was that the distance off bottom for acoustically tagged red snapper was negatively related to wave orbital velocity and current speed (Fig. 8), but there were three main differences. First, bottom temperature was excluded from the distance off bottom GAMM, whereas it was an important variable explaining red snapper movements. Second, fish were more likely to display a larger (~ 1 m) distance off bottom during the day compared to crepuscular periods or night, when they were 25% lower in the water column. Last, the distance off bottom for acoustically tagged red snapper was strongly and positively related to the upwelling index, with fish being approximately 3.5 m (240%) higher in the water column on average when the upwelling index was high compared to when it was low.

**Figure 9:**
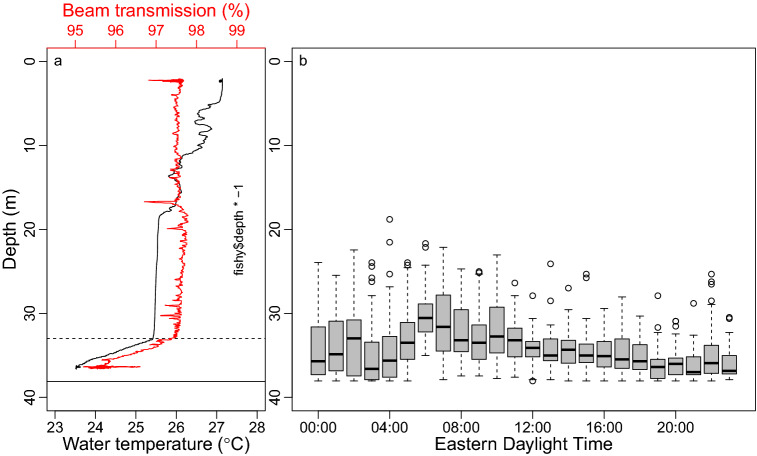
(**a**) Depth-specific water temperature (°C; black line) and beam transmission (%; red line) data from a conductivity-temperature-depth cast taken in the study area on 29 June 2019, showing the layer of upwelled water from the seafloor (~ 37.5 m; solid black horizontal line) up to approximately 33 m deep (horizontal dashed line). (**b**) Boxplot of red snapper (*Lutjanus campechanus*) depths binned by hour on the same day in which the conductivity-temperature-depth cast was taken (29 June 2019, 07:40 EDT). The thick horizontal lines are medians, boxes represent the first and third quartiles, whiskers are 1.5 times the interquartile range, and any depth estimates beyond the whiskers are shown by open points.

Depth-specific water temperature and beam transmission information collected at the study site on 29 June 2019 (07:40 EDT) indicated a layer of cold, turbid water extending approximately 5 m off the seafloor (~ 33 m deep; Fig. 9). The upwelled water was approximately 2 °C colder than the rest of the water column, beam transmission declined approximately 2% in the upwelled water as well, but dissolved oxygen was consistently high throughout the water column (i.e., 6.6–7.0 mg/L). Acoustically tagged red snapper present in the study area on 29 June 2019 (*N* = 9) were found a variety of distances off the bottom, generally being closer to the bottom during the night and farther off the bottom during the early morning and day (Fig. 9). When fish were found off the bottom, they were often located just above the upwelled water (i.e., 30–33 m deep), especially in the morning hours (0500–1200 Eastern Daylight Time).

## Discussion

We used a fine-scale acoustic telemetry positioning system to unravel the complex movements of red snapper at a natural hardbottom site in the coastal ocean. The spatial precision of our telemetry study was extremely high (< 2 m), suggesting that the observed movements of red snapper in our study were accurate and not the result of positional error. Red snapper movement rates and distance off bottom varied over diel cycles, with fish spending more time off the bottom and moving less during the day than night. Movement rates also varied by fish size, with larger fish spending time farther off the bottom than smaller fish. Moreover, water temperature had the largest influence on red snapper movements, with absolute bottom water temperature most strongly influencing movement rate and the difference in surface and bottom water temperatures driving their vertical distribution. Taken together, red snapper moved throughout their three-dimensional environment in complex ways and in response to multiple cues, with movement patterns variable across individuals, diel periods, and environmental conditions.

Most studies have shown that red snapper is a demersal fish species that displays a strong affinity for natural hardbottom or artificial structures^27,31^. However, our results mirror the findings of Williams-Grove and Szedlmayer^29^ that suggest red snapper make vertical movements into the water column – often well above structure – during late spring and summer months, indicating that such movements for the species may be common. Moreover, in our study and Williams-Grove and Szedlmayer^29^, when vertical movements occurred, they were more commonly observed during the day than at night. The underlying reasons for red snapper diel vertical migrations are unknown, but Williams-Grove and Szedlmayer^29^ suggest it may be related to spawning, low dissolved oxygen concentrations near the bottom^32^, foraging activity, or attraction to vessels^33^. In our study, the consistent movement of red snapper up into the water column primarily during the day and only when upwelling was occurring appears to be inconsistent with each of these hypotheses.

Diel variation in the distance off bottom and movement rate for red snapper may be related to their feeding mode and a thermoregulatory strategy to increase growth rate during cold-water upwelling events. All lines of evidence suggest that red snapper behave like most other lutjanids and feed more during crepuscular periods and at night than during the day^34,35^. Previous studies indicate that most prey consumed by red snapper are demersal^36–38^, and the highest proportion of red snapper stomachs containing prey occurred at night^36^. We also observed lower movement rates for red snapper during the day compared to at night, suggesting red snapper foraging rate declines during the day, which is consistent with Ouzts and Szedlmayer’s^36^ observation that gut fullness values were lowest at dusk. Taken together, it appears as though red snapper primarily forage near the seafloor during the night, and if they encounter cold water, they move vertically into warmer water (if present) to digest. It should be noted that the absolute bottom water temperature during periods of upwelling (~ 22–24 °C in May–July) were actually warmer than near the end of our study (< 22 °C in late November and December), so it appears to be the availability of warmer water higher in the water column—not the absolute bottom water temperature itself—that drives fish vertically. These results are similar to juvenile Bear Lake sculpin (*Cottus extensus*) that feed on benthic prey during the day, but migrate vertically at night into warmer water to aid digestion and thus increase growth rate (by approximately 300%) compared to individuals not migrating vertically^15^. Similar behaviors have been observed for sharks, except that they tend to migrate vertically at night to feed in warmer waters and digest in cooler, deeper waters during the day^16,19^. We are unaware of any examples in the literature of demersal reef fishes displaying vertical thermotaxis, which appears to be the case for red snapper in our study. Diel diet sampling and bioenergetics modeling of red snapper could be used to test this nascent hypothesis.

Red snapper movement rates were also related to water temperature, with fish moving more at lower compared to higher bottom water temperatures. Most fish (including red snapper) are ectothermic and the temperature of their bodies is typically close to that of the water^39^. In ectothermic fishes, swimming performance typically increases with water temperature, but eventually decreases when reaching the upper range of their thermal tolerance^40^. It is unlikely that the highest bottom water temperatures observed in our study (~ 28 °C) approached the upper range of red snapper thermal tolerance given that North Carolina is in the northernmost range of the species, suggesting other factors may explain the negative relationship. In colder water, red snapper may have to search greater distances for prey or sufficiently warm water. For instance, during large upwelling events during summer on the east coast of Florida, red snapper may evacuate the area to avoid bottom upwelling of cold water^41,42^. Our study also did not occur during the coldest months of the year (i.e., January–March), so the negative relationship we observed between movement rate and water temperature may not be observed if winter data were included (e.g., Ref.^29^).

Red snapper were also influenced by the movement of water at the seabed. In our study, we tested for the influence of two types of water movement on red snapper movements: current speed, which was the continuous horizontal movement of water at the seabed as determined by current probes, and wave orbital velocity, which was the wave-generated oscillatory water flow (“sloshing”) at the seabed typically associated with storms. Red snapper were somewhat less likely to move, and spent more time closer to the bottom, when the movement of water increased. This result is exactly the opposite found for gray triggerfish, which greatly increased their movement and emigration rates during storms when wave orbital velocities were high^43^. With a larger mean body size and a different body morphology, red snapper may be better equipped to deal with storm effects than the generally smaller gray triggerfish^42^. Even during Hurricane Dorian on 6 September 2019 that brought 8.1 m surface waves to the study area, acoustically tagged red snapper movement rates were mostly unaffected, which adds to a conflicting body of research examining storm effects on red snapper (see Ref.^44^ for a review).

Red snapper movement rates and distance off bottom varied substantially across individuals tagged in our study. There was a 535% difference in movement rates from individuals moving the least to those moving the most in our study, and a 1330% difference in distance off bottom among individuals, even though fish size only explained a modest amount of variation in these two variables. Had we been able to tag a broader size distribution of red snapper in our study, it is possible that size may have explained more of the variability in movement rates or distance off the bottom. Larger red snapper displayed higher movement rates and greater distance off bottom compared to smaller fish. This result is consistent with most previous work that has found a positive relationship between movement rates and animal size, perhaps due to increased absolute food requirements and reduced predation risk as body size increases^43,45^. However, there was also substantial variability among individuals of the same size, suggesting other traits like sex or personality traits (e.g., bold versus shy) may contribute to differences in movement patterns^3,46^. Unfortunately, sex of red snapper cannot be determined externally.

Despite showing that red snapper movement rates and distance off bottom varied across individuals, diel periods, and environmental conditions, our models only explained a modest amount (5–30%) of the total variability observed in our two response variables. Therefore, 70–95% of the variability in movement rates or distance off bottom remained unexplained, suggesting unmeasured variables are important in influencing red snapper movements or movement rates for fishes are inherently stochastic. Potential unmeasured variables include predator avoidance, prey distribution and abundance, social interactions, habitat characteristics, environmental variables not included in the current study, and positional error of the Vemco positioning system (VPS). Because the distance off bottom models explained more variability than movement rate models, our results suggest that red snapper horizontal movements may be more complicated and difficult to explain than their vertical movements.

The fine-scale acoustic positioning system we employed could be used to address a variety of questions concerning the three-dimensional movements and space use of demersal marine and freshwater fishes. VPS has been used effectively to quantify habitat use^47^, mortality rates^47^, bait responses^48^, and movements^4,27–29,43^ of various species of fishes and invertebrates, and wider use of this methodology would improve our knowledge of fish movements. It was also informative to deploy a reference transmitter in the study system to estimate the accuracy and precision of fish positions, and we recommend future VPS studies do the same. VPS studies will be especially effective for species that exhibit high site fidelity, retain tags, and survive the tagging process in areas with high transmitter detection probability^50^. Our primary analysis employed GAMMs to analyze the hourly movement rates and distance off bottom of red snapper from VPS, with the chief benefit being the ability to assess the influence of multiple predictor variables on red snapper movements while simultaneously accounting for temporal autocorrelation in the data.

Our results have implications for scientific surveys of red snapper used for population assessment and management. For instance, the likelihood of catching red snapper (i.e., catchability) may decline during upwelling events because their metabolic rate is reduced in cold water, but also because red snapper may be found higher up in the water column, farther away from sampling gears that typically rest on the bottom^51^. If not accounted for, this phenomenon may result in the appearance of red snapper evacuating an area of upwelling when in fact they may have simply moved into waters above it.

There were several shortcomings of our study. First, we excluded individual movement rate estimates for any fish calculated from two detections occurring over a time period greater than 20 min, because previous research has found an increasing negative bias of movement rates when the time interval in tracking studies increased^52^. However, this also resulted in the exclusion of periodic large movements of fish leaving from, or returning to, the study area, suggesting that in some cases our movement rate estimates may be biased low. Second, our study was correlational in nature, so causation could not be determined. Last, we would have preferred to use movement rate or distance off bottom estimates from individual fish in an integrated GAMM analysis (e.g., including unique fish as a random effect), but those models did not converge due to large sample sizes, so we necessarily used mean hourly values instead.

We have entered a golden age of animal tracking science, where technological advances lead to new and cheaper approaches that can collect precise, fine-scale spatial positions, and new analytical methods that can be used to analyze those data^53^. More species can now be tracked than ever before given recent efforts to make tags smaller, cheaper, more reliable, and less invasive, and the number of ecological questions that can be addressed using telemetry has likewise increased concomitantly. However, there is still much to learn about how oceanography, habitat, ecomorphology, feeding mode, and social interactions combine to determine the causes and consequences of fish movement. Movement is a central organizing feature of the ecology of fish, and elucidating when, where, why, and how fish move will benefit their sustainable management.

## Methods

### Study site

This study took place at a temperate reef called the “Chicken Rock” in waters off the coast of North Carolina, USA, between Cape Hatteras and Cape Lookout (Raleigh Bay; Fig. 1). The seafloor of the Chicken Rock is composed of low-relief hardbottom and sand. The Chicken Rock is approximately 37 m deep (Fig. 2) and is an ideal location for this study for three reasons. First, it has a relatively flat seafloor that allows for a high detection rate of acoustically tagged fish^49^. Second, a high-resolution bathymetric map was available for the area (C. Taylor, National Centers for Coastal Ocean Science). Third, many red snapper occupy the area, allowing us to catch and tag fish relatively easily. Recreational and commercial fishing occurs at the Chicken Rock year-round for a variety of species, but red snapper can only be retained during short open seasons that have occurred periodically since 2010.

### Data collection

We quantified the fine-scale movements and distance off bottom for red snapper using VPS (Innovasea, Nova Scotia, Canada). VPS uses a time-difference-of-arrival algorithm to determine the location of coded acoustic transmitters that have been detected by at least three submersible acoustic receivers^50^. Highly precise fish positions (~ 1 m resolution) are possible if time is synchronized exactly across all receivers, which is accomplished by using sync tags that are either deployed independently throughout the receiver array or built into the receivers themselves. One downside of VPS is that data are not available in real time; receivers must be physically recovered to download data, and then data have to be sent to Vemco to determine fish positions. The advantages of VPS, however, are immense, especially in providing highly precise spatial positions each time acoustic signals are emitted from transmitters. VPS has been used many times to successfully quantify demersal fish movements^27,28,49,50,54^, and three-dimensional movements can be determined if pressure sensors are built into transmitters^23,42^.

We deployed an array of 20 submersible VR2AR receivers at the Chicken Rock on 17 April 2019. Receivers were deployed in three rows of seven receivers, except for a single receiver in the northeast corner of the grid. Based on previously estimated detection distances of 200–400 m^49,55^, receivers were separated 200 m from each other, so the entire receiver grid occupied an area of approximately 400 × 1200 m (0.48 km^2^; Fig. 2). Each receiver was connected to a line between a 36-kg steel weight and a 28-cm diameter plastic float with 8.8 kg of buoyancy, with each receiver positioned approximately 3 m off the seafloor. Each VR2AR included its own sync tag for time synchronization and acoustic release so receivers could be retrieved at the end of the study. A TCM-1 current probe (Lowell Instruments) was attached to each of three receiver buoys spread out across our receiver array (Fig. 2) to collect minute-by-minute current speed and bottom water temperature.

We also deployed a reference transmitter (Vemco V13T-1x) in the receiver array on 17 April 2019 (Fig. 2) to calculate sound speed velocity for VPS analyses and quantify positional error of transmitters in the receiver array by comparing its known location to its estimated positions over the course of the study. The reference transmitter was connected to a line with a weight at one end and a buoy at the other, had a 550–650 s random ping interval, and operated on a frequency of 69 kHz.

A total of 44 red snapper were tagged in this study. Twenty-three red snapper were tagged on 7 May 2019, nineteen were tagged on 13 August 2019, one was tagged on 30 August 2019, and one was tagged on 22 September 2019 (Table 1). Most of these red snapper (*N* = 43) were caught via hook-and-line using either circle or J-style hooks, but one red snapper (tagged on 30 August 2019) was caught in a baited fish trap. Fish in good condition (i.e., no visible signs of barotrauma, jaw hooked, active) were tagged externally because external attachment is fast (i.e., greatly reducing surface time^56^) and externally attached transmitters are detected better than surgically implanted transmitters^57^. The downside is that transmitter retention is typically lower for externally attached transmitters compared to surgically implanted transmitters.

We tagged red snapper with Vemco V13P-1 × transmitters that were 13 mm wide, 46 mm long, weighed 13 g in air, had a 130–230 s pulse interval, a 613 d battery life, and operated on a frequency of 69 kHz. Each transmitter also contained a pressure sensor, which was used to determine the depth of fish for each acoustic signal (accuracy = 1.7 m). Before field work began, stainless steel wire (0.89-mm diameter) was wrapped around the non-transmitting end of the transmitter, glued with marine adhesive (3 M 5200), and covered in heat shrink tubing. Approximately 15 cm of stainless steel wire that extended beyond the transmitter was straightened, and the end was sharpened.

Upon capture, red snapper had their head and eyes covered in a wet towel and were measured for total length (mm). The sharpened transmitter wire was inserted laterally through the dorsal musculature of the fish approximately 2.5 cm posterior to, and 2.5 cm below, the insertion of the fish’s first dorsal spine. The wire was pushed laterally through the fish until the transmitter was pulled firmly against the fish’s left side, while the sharpened end emerged from the same spot on the right side of the fish. An aluminum washer was threaded onto the protruding wire, followed by a #1 double sleeve steel crimp, which was crimped onto the wire once the washer and crimp were held firmly on the right side of the fish. The wire beyond the crimp and wet towel were removed, the fish was attached to a weighted SeaQualizer fish release tool, and the fish was descended to a depth of approximately 31 m before being released by the device. The total surface time for each tagged red snapper was approximately 1.5 min.

### Data analyses

We first assessed whether potential error in red snapper positions could influence study results. For each reference tag position estimated by VPS, we calculated horizontal positional error as the difference between the known reference tag location and its estimated position based on VPS. We visualized daily horizontal positional error of the reference transmitter with a boxplot. Daily values were provided to determine if any changes in positional error occurred over time.

Next, we used positional and depth data from fish that were monitored to determine the fate of each individual and classified them based on four events: tag loss, emigration, harvest, or predation^48^. Fish were assumed to have lost transmitters if the transmitter stopped moving; they were assumed to have emigrated if the transmitter moved to the edge of the receiver array before disappearing. Harvest was assumed if fish disappeared from within the receiver array. Predation (e.g., by sharks) was inferred from VPS data in one of three ways: (1) transmitters moved horizontally much faster than normal red snapper swimming speeds, (2) transmitters moved quickly across a wide range of depths, typically from the bottom to the surface and back, and (3) a reduced frequency of detections, as might be expected for transmitters in the abdominal cavity of a shark. VPS data were censored after the point at which any fish experienced tag loss, harvest, or predation, and only fish with 100 or more spatial positions were included in the analyses.

We then estimated movement rates of each fish over time. Movement rate (m s^−1^) was quantified as the distance moved between each successive pair of spatial positions divided by the time between detections. One challenge with using movement rates is that straight-line movements are assumed between detections, when in reality fish may not move in straight lines. Red snapper were detected on average every 2–4 min, so this issue is less of a problem in our study compared to those using longer time intervals between detections^51^, but our movement rates can be considered minimum estimates. To further prevent negatively biased movement rate estimates, we excluded movement rate estimates for time intervals longer than 20 min; this decision had negligible effects on results (see *Discussion*).

We also estimated the distance off the seafloor for all detections of acoustically tagged red snapper. We calculated distance off the bottom (m) for each fish position as the depth of the seafloor at that location minus the depth of the fish. We encountered an issue with some transmitters after tag loss whereby depth readings appeared to slowly drift towards shallower readings even though the transmitter was sitting on the bottom and not moving horizontally; in a few instances, this same depth drift issue was detected for transmitters attached to fish alive in the study area (i.e., distance off bottom was greater than zero for long periods of time, which never occurred for red snapper with working pressure sensors). We do not know the reason for these rare instances of depth drift by the pressure sensors, but out of caution we censored depth data for fish whose transmitters provided dubious depth data.

We evaluated whether individual differences in movement rates or distance off the bottom were apparent. We created boxplots of movement rate and distance off bottom for each fish in the study, and tested for differences among individuals using a linear model where fish number was included as a categorical variable. We compared the Akaike information criterion (AIC) values of models including fish number with an intercept-only model where fish number was excluded, and models with the lowest AIC value (ΔAIC = 0) were considered the most parsimonious formulations^58^. Movement rate was positively skewed, so it was log-transformed to improve model fit. Model diagnostics (i.e., quantile–quantile, histogram of residuals, residuals versus linear predictions, response versus fitted values plots) were used to confirm that final models met assumptions of equal variance and normal residuals. We used R version 3.6.3^59^ to carry out all statistical tests and to create all figures.

Ideally, we would then test for the effects of environmental conditions and fish size on red snapper horizontal and vertical movements using a single, integrated analysis. However, models accounting for temporal autocorrelation and incorporating individual movement rate estimates from each fish as the response variable (i.e., including fish number as a random effect) did not converge, possibly due to large sample sizes (*N* = 346,363), so we used mean hourly values instead. The downside of this approach is that fish size had to be evaluated separately from the effects of environmental conditions, as described below. However, note that covariate relationships changed very little across a wide variety of model formulations.

We tested for the effects of fish size on movement rate and distance off the bottom using generalized additive models^60^ (GAMs). GAMs are a regression modeling approach that relate a response variable to a single or multiple predictor variables using nonlinear, linear, or categorical functions. Mean log-transformed movement rate or distance off bottom were the response variables of these models and cubic-spline-smoothed fish total length (mm) was included as the predictor variable. As above, we compared the AIC values of models including fish size with an intercept-only model where fish size was excluded, and the model with the lowest AIC value was selected as the best model.

We then assessed the influence of various environmental factors (see below) on red snapper movement rate and distance off bottom using GAMs. For these analyses, choosing the appropriate time scale for binning response and predictor data was critical. Longer time steps (i.e., day) were problematic because response and predictor variables frequently varied over much shorter time frames, while extremely short time steps (i.e., minute) were often lacking response and predictor variable information. Therefore, we used an hourly time step for this procedure. The main concern of using an hourly step is that any particular hourly time bin is likely to be more similar to the time bin nearest in time compared to a randomly selected time bin; in other words, time bins are not truly independent of one another^61^ (i.e., data are temporally autocorrelated). Not accounting for temporal autocorrelation that is present often leads to a negative bias in estimated regression coefficients and confidence intervals. To account for temporal autocorrelation, we used generalized additive mixed models (GAMMs) that included an autoregressive term for model errors. We used a likelihood ratio test to compare our GAMM to a GAM that did not include autoregressive errors, and in both cases GAMMs were selected over GAMs so they were used for movement and distance off bottom models.

We limited our GAMMs to five predictor variables based on previous work. The first predictor variable was time of day, which we included because red snapper movements have been shown to vary over diel periods^29^. We included time of day (*tod*) as a categorical variable with three levels: day, crepuscular period, and night. Because sunrise and sunset times varied over the course of our 8-mo study, we defined crepuscular periods as a one hour period of time spanning 30 min before sunrise or sunset to 30 min after sunrise or sunset for each day of the study. Day was defined as 30 min after sunrise to 30 min before sunset, and night was defined as 30 min after sunset to 30 min before sunrise.

Bottom water temperature has been shown to be strongly correlated with red snapper movements and home range size^28,29^, so it was included as our second predictor variable. We calculated bottom water temperature (*temp*; °C) as the mean bottom temperature measured across the three current probes deployed in the receiver array. Cold bottom water temperatures were observed near the conclusion of our study (December 2019) due to declining air temperatures and water column mixing, but also during periodic upwelling events that occurred from late May through early August. Upwelling is a common oceanographic feature of the region, occurring when upwelling-favorable winds are observed concurrent with the Gulf Stream being in a relatively inshore position^62,63^. Upwelled water that is cold and nutrient-rich is generally only found near the bottom, which tends to cause phytoplankton blooms near the bottom that decrease water clarity. From preliminary analyses of red snapper VPS data, we observed differing behaviors of fish during periods of upwelling than periods lacking upwelling. Therefore, we developed an upwelling index as our third predictor variable, which was calculated as the difference between the surface water temperature and mean bottom water temperature (*upwel*; °C). Surface water temperature was not available at the study site, so we obtained hourly surface temperature data from the nearest NOAA buoy (#41159), which was located ~ 85 km southwest of the study site in a similar water depth (Fig. 1). We assume that surface water temperature at the study site could be approximated with data from this buoy, which is a reasonable assumption given surface water temperature and wave heights from this buoy were strongly correlated with values from another buoy (NOAA buoy #41025) ~ 70 km northeast of the study site.

The last two predictor variables involved properties of water movement at the seafloor in the study area. The fourth predictor variable was wave orbital velocity (*wov*; m s^−1^), which is a measure of the wave-generated oscillatory flow (“sloshing”) of water at the seabed. Wave orbital velocity was included because it was much more strongly correlated with gray triggerfish (*Balistes capriscus*) movement rates at the Chicken Rock area than either barometric pressure or bottom water temperature^43^, the latter of which have been shown to be more important for organisms in shallow water^64,65^. Wave orbital velocity was calculated following Bacheler et al.^43^ using the properties of surface wave period and height, which were also obtained from NOAA buoy 41159. The last predictor variable included in models was current speed (*cur*; cm s^−1^), which was calculated as the mean horizontal current speed from the three current probes deployed on the bottom in the receiver array.

The GAMMs were formulated as:

1 $$y = \upalpha + f(tod) + s_{1} (temp) + s_{2} (upwel) + s_{3}(wov) + s_{4} (cur) + \varepsilon ,$$

where y is either acoustically tagged red snapper log-transformed movement rate (m s^−1^) or distance off the bottom (m), α is the intercept, *f* is a categorical function, *s*_1-4_ are cubic spline smoothing functions, and $\varepsilon$ is the autoregressive error term accounting for temporal autocorrelation in the data.

We employed model selection techniques to assess the importance of predictor variables. Specifically, we compared full models that included all five predictor variables to reduced models that included fewer predictor variables. Model comparisons were made using AIC, and models with the lowest AIC value (ΔAIC = 0) were again considered the most parsimonious. Various diagnostics of final models were examined using the “gam.check” function in the mgcv library to ensure model fit was suitable.

Given the importance of upwelling to the vertical movements of red snapper (see *Results* section), we last include results from a conductivity-temperature-depth (CTD) cast taken in the study area from the NOAA Ship *Pisces* on 29 June 2019 (07:40 EDT), which occurred during a time when bottom upwelling was present. This CTD cast was conducted using a Sea-Bird SBE 9 deployed from the surface to within 1.5 m of the bottom, and depth-specific water temperature and beam transmission data were provided to highlight the vertical extent of upwelling on this particular day. Beam transmission is the fraction of a light source reaching a light detector set a distance away and is a quantitative measure of water clarity; a common feature of upwelling in the region (in addition to cold water) is declining clarity due to increased production within nutrient-rich, upwelled water near the bottom. We combine these water temperature and beam transmission data with a boxplot of red snapper distances off the bottom by hour throughout the same day the CTD cast was taken (29 June 2019).

### Ethics approval

The tagging protocol was approved by the Institutional Animal Care and Use Committee (# NCA19-002) of the North Carolina Aquariums on 20 March 2019. All research activities were carried out under a Scientific Research Permit issued to Nathan Bacheler on 10 April 2017 by the Southeast Regional Office of the U.S. National Marine Fisheries Service, in accordance with the relevant guidelines and regulations on the ethical use of animals as experimental subjects.

## Data Availability

Due to ethical concerns about making the precise locations of red snapper available to the public, data are available upon reasonable request to the corresponding author (nate.bacheler@noaa.gov).
